# Reprogramming fibroblasts and peripheral blood cells from a *C9ORF72* patient: A proof‐of‐principle study

**DOI:** 10.1111/jcmm.15048

**Published:** 2020-03-03

**Authors:** Donatella Bardelli, Francesca Sassone, Claudia Colombrita, Clara Volpe, Valentina Gumina, Silvia Peverelli, Ilaria Catusi, Antonia Ratti, Vincenzo Silani, Patrizia Bossolasco

**Affiliations:** ^1^ Department of Neurology and Laboratory of Neuroscience Istituto Auxologico Italiano IRCCS Milan Italy; ^2^ “Aldo Ravelli” Center for Neurotechnology and Experimental Brain Therapeutics Università degli Studi di Milano Milan Italy; ^3^ Department of Medical Biotechnology and Translational Medicine Università degli Studi di Milano Milan Italy; ^4^ Lab. di Citogenetica Medica IRCCS Istituto Auxologico Italiano Milano Italy; ^5^ Dino Ferrari” Center Department of Pathophysiology and Transplantation Università degli Studi di Milano Milan Italy; ^6^ Present address: Axxam Spa OpenZone Bresso (Milan) Italy

**Keywords:** amyotrophic lateral sclerosis, *C9ORF72*, fibroblasts, iPSCs, motor neuron, peripheral blood cells, repeat expansion, reprogramming, RNA foci, TDP‐43

## Abstract

As for the majority of neurodegenerative diseases, pathological mechanisms of amyotrophic lateral sclerosis (ALS) have been challenging to study due to the difficult access to alive patients' cells. Induced pluripotent stem cells (iPSCs) offer a useful in vitro system for modelling human diseases. iPSCs can be theoretically obtained by reprogramming any somatic tissue although fibroblasts (FB) remain the most used cells. However, reprogramming peripheral blood cells (PB) may offer significant advantages. In order to investigate whether the choice of starting cells may affect reprogramming and motor neuron (MNs) differentiation potential, we used both FB and PB from a same *C9ORF72*‐mutated ALS patient to obtain iPSCs and compared several hallmarks of the pathology. We found that both iPSCs and MNs derived from the two tissues showed identical properties and features and can therefore be used interchangeably, giving the opportunity to easily obtain iPSCs from a more manageable source of cells, such as PB.

## 1 INTRODUCTION

Amyotrophic lateral sclerosis (ALS) is a devastating and fatal neurodegenerative disease. This disorder is characterized by a progressive motor neuron (MNs) loss in brain and spinal cord, causing paralysis, respiratory failure and death within 5 years from the diagnosis.^1^ At molecular level, the DNA/RNA‐binding protein TAR (trans‐activation response element) DNA‐binding protein (TDP‐43) was described to be the major component of the pathological aggregates found in brains of ALS patients.^2^ The presence of the GGGGCC (G_4_C_2_)‐repeat expansion in the noncoding region of the open reading frame 72 (*C9ORF72*) gene on the chromosome 9 is the most frequent genetic cause of ALS and frontotemporal dementia (FTD). Usually, in healthy subjects, the number of G_4_C_2_‐repeats is lower than 30 but conversely can be in the order of thousands in ALS/FTD patients. The *C9ORF72* repeat expansion determines several pathological hallmarks, among which there is a toxic gain of function of RNA repeats, accompanied by foci formation. No effective cure for this disease is yet available, mainly due to the fact that pathological mechanisms are difficult to study owing to the impossibility to obtain affected cells from alive patients. In addition, *post mortem* brain tissues represent only the end stage of the disease limiting the comprehension of cellular and structural defects leading to the onset of neurodegeneration. Studies to elucidate ALS pathological mechanisms have also been limited by the lack of models able to fully mimic affected MNs until the advent of induced pluripotent stem cells (iPSCs).^3^ This powerful in vitro model allows the generation of patient‐specific cell lines that can be differentiated into MNs, affected in ALS. iPSCs can be obtained by reprogramming different cell types such as CD34^+^ cord blood cells,^4^ keratinocytes^5^ or peripheral blood (PB),^6^, ^7^ but fibroblasts (FB) remain the most widely used.^8^, ^9^, ^10^, ^11^ However, FB do not necessarily represent the best cell source to generate iPSCs, not even displaying the highest reprogramming efficacy and needing in vitro passages before reprogramming increasing the risk to accumulate genetic alteration. Additionally, if compared to PB, a biopsy punch is doubtless more invasive than a venipuncture. PB has been already shown to be an easily accessible source of patient tissue without the need to extensively maintain cells in culture prior to reprogramming experiments.^6^ Furthermore, using PB as starting source may allow to achieve longitudinal studies of the same patient over many years and easily create a bio‐bank of collected samples. In this work, we investigated and compared the key aspects of iPSCs and iPSC‐derived MNs obtained from FB and PB of a *C9ORF72*‐mutated patient.

## 2 MATERIAL AND METHODS

This study was approved by the ethics committee of IRCCS Istituto Auxologico Italiano.

### 2.1 Sample collection and processing

Skin biopsy and PB were collected from a 49 years old female affected by ALS and carrying a *C9ORF72* repeat expansion. Written informed consent was obtained from the patient. FB were obtained from skin biopsy as follow: the skin tissue fragment was transferred in a 60 mm culture dish and subcutaneous residuals removed. Few drops of FB medium (RPMI 1640 (Euroclone) supplemented with 10% foetal bovine serum (FBS, Sigma‐Aldrich), 2 mmol/L l‐glutamine, 2.5 μg/mL amphotericin B (Sigma‐Aldrich), 100 U/mL penicillin and 100 μg/mL streptomycin) were added, and the sample was allowed to adhere to the bottom of the dish overnight. The following day, fresh medium was added and changed twice a week after attachment and outgrowth of cells from skin dissection. FB were maintained in medium and expanded at 37°C with 5% CO_2_. Reprogramming was performed by the fifth passage. Peripheral blood mononuclear cells (PBMCs) were isolated by layering diluted blood sample on a density gradient (1.077 g/mL) (Histopaque^®^‐1077, Sigma‐Aldrich) followed by centrifugation. PBMCs layered in the plasma‐density medium interface were washed, counted and cryopreserved in FBS/10% DMSO until reprogramming.

### 2.2 Reprogramming of iPSCs

iPSCs were obtained using the CytoTune^®^‐iPS 2.0 Sendai Reprogramming Kit (Thermo Fisher Scientific) by adding the Klf4, Oct4, Sox2 and c‐Myc virus, following manufacturer's instructions. Briefly, 2 days before reprogramming, FB were seeded in a well of a 6‐well plate in FB medium at 30%‐60% confluence. After reprogramming, they were maintained in FB medium until day 7 and therefore plated onto Matrigel (Corning) coated dishes to allow colony attachment. For PB, 500 000 PBMCs were seeded in a 24‐well plate and cultured with the addition of IL‐3 (20 ng/mL), IL‐6 (20 ng/mL), SCF (100 ng/mL) and FTL‐3 ligand (100 ng/mL) (all from Gibco) in StemPro‐34 medium (Thermo Fisher Scientific) for 4 days. The third day after transduction, cells were transferred on Matrigel‐coated dishes and cultured in StemPro‐34 without cytokines.

For both samples, the eighth day after reprogramming, medium was replaced with the iPSCs‐specific Essential 8 medium (Thermo Fisher Scientific). Spent medium was daily replaced with fresh medium and the cultures monitored for the emergence of iPSC colonies. When colonies reached the appropriate size, six clones per sample were picked and transferred on new Matrigel‐coated dishes for expansion. Colonies, passaged using an EDTA 0.5 mmol/L solution, were expanded in Essential 8 medium for at least six passages before being characterized and differentiated.

### 2.3 Characterization of iPSCs clones

After at least six passages in culture, karyotyping and characterization were performed on clones from each starting material.

#### 2.3.1 Karyotyping

Colcemid solution (KaryoMAX™, Thermo Fisher Scientific) was added overnight to cultured cells in logarithmic phase. Chromosomes were stained with the fluorescent dye quinacrine mustard (Sigma‐Aldrich). Q‐Band stained chromosomes were analysed.

#### 2.3.2 Repeat primed PCR

The maintenance of repeat expansion was evaluated by repeat primed PCR as previously described.^12^ DNA was extracted from reprogrammed cells using Wizard^®^ Genomic DNA Purification Kit (Promega Corporation, Madison, WI). High‐quality genomic DNA (200 ng) was amplified using three specific primers (1.4 µmol/L FAM forward primer, 0.7 µmol/L Reverse primer, 1.4 µmol/L Reverse Anchor M13 primer [see Appendix S1]) and 180 µmol/L of deAZAdGTP/dNTPs mix (Roche). The PCR reaction was performed as here described: initial denaturation at 98°C for 10 minutes followed by 10 cycles of denaturation at 97°C for 35 seconds, annealing at 64°C for 2 minutes and extension at 68°C for 8 minutes. Subsequently, additional 25 cycles with denaturation at 97°C for 35 seconds, annealing at 64°C for 2 minutes and extension at 68°C for 8 minutes adding 20 seconds to each additional cycle were performed. After amplification, 1.5 µL of the PCR product was mixed with 15.5 µL Hi‐Di™ Formamide (Applied Biosystems) and 0.5 µL GeneScan™ 500 ROX™ dye Size Standard (Applied Biosystems). The capillary electrophoresis was performed on the ABI 3500 Genetic Analyzer (Applied Biosystems), and output was analysed with Gene Mapper v.4 software (Applied Biosystem).

#### 2.3.3 Stemness evaluation

For reverse transcription (RT), total RNA was isolated from both iPSC clones and starting tissues using Trizol Reagent (Thermo Fisher Scientific) following manufacturer's instruction and reverse transcribed into cDNA using SuperScript II reverse transcriptase (Thermo Fisher scientific). Briefly, RT was primed with Oligo(dt) (Eurofins Genomics, Luxembourg, LU) and reaction incubated at 42°C for 40 and 15 minutes at 70°C. Loss of SeV genome and viral transgenes was assessed by RT‐PCR (denaturation 95°C 30 second, annealing 54°C 30 second, elongation 72°C 1 minutes, for 35 cycles) using commercial primers (Thermo Fisher Scientific, see Appendix S1). RT‐PCR was then performed (denaturation 95°C 30 seconds, annealing 60°C 30 seconds, elongation 72°C 1 minutes, for 35 cycles) to reveal expression of stemness markers, using the following primers: Sox 2, Oct 3/4 and Nanog (see Appendix S1).

For immunofluorescence, iPSCs cultured on coverslips were fixed in 4% paraformaldehyde (Santa Cruz Biotechnology) for 20 minutes and permeabilized with 0.3% Triton X‐100 (Sigma‐Aldrich). Cells were then incubated for 20 minutes in 10% normal goat serum (NGS) (Gibco) in PBS and primary antibodies added for 90 minutes at 37°C. After two washes with 10% NGS in PBS, cells were stained with Alexa 488 and Alexa 555 conjugated secondary antibodies (Invitrogen, Thermo Fisher Scientific, Waltham, MA, USA). Nuclei were stained using 4′,6‐diamidino‐2‐phenylindole, dihydrochloride (DAPI) (Sigma‐Aldrich) and images acquired with a confocal microscope (Nikon Eclipse C1, Minato). The following primary antibodies were used: TRA‐1‐60 (1:125, Invitrogen), SSEA4 (1:100, Invitrogen) and alkaline phosphatase (1:250, Abcam).

#### 2.3.4 In vitro spontaneous differentiation

Embryoid bodies (EBs) were generated by suspending iPSCs in low adhesion plates in HuES medium (DMEM/F12, 20% knock‐out serum replacement, 2 mmol/L l‐glutamine, 10 U/mL penicillin, 10 μg/mL streptomycin, 0.1 mmol/L MEM NEAA, 110 μmol/L β‐mercaptoethanol [all from Thermo Fisher Scientific]). After 7 days, EBs were plated on Matrigel‐coated coverslips and grown in Essential 8 medium for additional 10 days. Expression of the three germ layer‐specific markers was evaluated by immunofluorescence (β III tubulin (1:500, Abcam) for ectoderm, desmin (1:10, Chemicon, Merck Millipore) for mesoderm and alpha‐fetoprotein (1:125, Invitrogen) for endoderm) as previously described.

### 2.4 Differentiation of iPSCs into neural stem cells (NSCs)

iPSCs were cultured in Induction medium (Neurobasal and 2% Neural Induction Supplement [Thermo Fisher Scientific]) for 7 days. On day eight, cells were harvested by Accutase (Euroclone) and seeded on Matrigel‐coated dishes in Expansion medium (50% Neurobasal, 50% DMEM/F12 and 4% Neural Induction Supplement) with addition of 10 μmol/L RHO‐associated kinase (ROCK) inhibitor Y27632 (Selleckchem). Cells were allowed to grow and expanded for at least five passages, until they showed a homogeneous neural morphology. Expression of NSCs‐specific marker nestin (1:100, Chemicon) was assessed by immunofluorescence.

### 2.5 Differentiation of iPSCs into MNs

MNs were differentiated as previously described.^13^ Briefly, iPSCs were grown in 100 mm dishes until confluence, harvested and placed into low adhesion dishes to generate EBs. Cells were grown in suspension for the first 2 days in HuES medium supplemented with 20 ng/mL basic fibroblast growth factor (Peprotech) and 20 μmol/L ROCK in order to enhance single cell survival. Neuralization was induced starting from the third day by adding 10 μmol/L SB431542 (Tocris) and 0.2 μmol/L LDN193189 (Sigma‐Aldrich) to the culture. The fourth day, EBs were switched to neural induction medium (DMEM/F12, 2 mmol/L l‐glutamine, 10 U/mL penicillin, 10 μg/mL streptomycin, 0.1 mmol/L MEM NEEA, 2 μg/mL heparin (Sigma‐Aldrich), 1% N2 supplement [Thermo Fisher Scientific]), supplemented with 20 μmol/L ROCK inhibitor, 0.4 μg/mL ascorbic acid (AA) (Sigma‐Aldrich), 1 μmol/L retinoic acid (RA) (Sigma‐Aldrich), 10 ng/mL brain‐derived neurotrophic factor (BDNF) (Peprotech). SB431542 and LDN193189 were added until day 7 when cultures were supplemented with 1 μmol/L smoothened agonist (Tocris) and 0.5 μmol/L purmorphamine (Sigma‐Aldrich). EBs were grown for 10 days with a medium change every other day. For dissociation of EBs, on day 17, 0.05% trypsin (Sigma‐Aldrich) was used and cells were plated on poly‐lysine (Sigma‐Aldrich)/laminin (Thermo Fisher Scientific)‐coated coverslips in 24‐well plates at a concentration of 5 × 10^5^ cells/well. Cells were cultured in neural differentiation medium (Neurobasal, 2 mmol/L l‐glutamine, 10 U/mL penicillin, 10 mg/mL streptomycin, 0.1 mmol/L MEM NEAA, 1% N2 supplement), supplemented with 2% B27 (Gibco), 1 μg/mL laminin, 25 μmol/L glutamate (Sigma‐Aldrich), 0.4 μg/mL AA, 10 ng/mL glial‐derived neurotrophic factor (GDNF) and 10 ng/mL ciliary neurotrophic factor (CNTF) (both from Peprotech). MNs were fixed for immunofluorescence or used for RNA/protein extraction after 10 days of differentiation. For immunofluorescence, the following MNs markers were used: β III tubulin, SMI312 (1:1000, Covance) and HB9 (1:100, DSHB, NIH) as previously described for the characterization of iPSCs.

RT‐PCR was performed as previously described to identify expression of Chat and HB9 markers (see Appendix S1).

### 2.6 TDP‐43 localization

Immunofluorescence analysis was performed as previously described for the characterization of iPSCs and MNs, using an anti‐TDP‐43‐2AP (1:500, Proteintech) and an anti‐phospho TDP‐43 (pS409/410) (1:200, Cosmobio) primary antibodies.

### 2.7 Western Blot

Cells collected by centrifugation and washed with ice‐cold PBS were lysed in lysis buffer (20 mmol/L Tris–HCl pH 7.5, 150 mmol/L NaCl, 1 mmol/L EDTA, 1 mmol/L EGTA, 1% Triton X‐100, protease inhibitor cocktail [Roche]). Cell extracts were quantified using Pierce BCA protein assay (Thermo Fisher Scientific) and 20 μg protein separated by SDS‐PAGE on 10% NuPAGE Bis‐Tris pre‐cast polyacrylamide gels (Thermo Fisher Scientific) and then transferred to nitrocellulose membranes. Membranes were blocked with 5% (w/v) nonfat dry milk in Tris‐buffered saline with Tween 20 (Sigma‐Aldrich) (TBST; 20 mmol/L Tris‐HCl [pH 7.6], 0.1% Tween 20, 137 mmol/L NaCl) for 1 hour. After blocking, membranes were incubated with primary antibodies (TDP‐43‐2AP (1:1000) and GAPDH [1:2000, Santa Cruz]) in blocking solution overnight at 4°C. After three washes, membranes were incubated with secondary antibodies (anti‐mouse/rabbit HRP‐conjugated, 1:10 000, both from Santa Cruz) for 1 hour at room temperature. The Novex ECL kit (Thermo Fisher Scientific) was used for detection of the HRP‐conjugated secondary antibody. Densitometric analyses were performed using ImageJ software (NIH).

### 2.8 RNA foci identification

RNA FISH was performed using 5′ a TYE‐563‐labelled locked nucleic acid (LNA) probe complementary to the sense RNA foci containing GGGGCC‐repeats (Exiqon Qiagen, Hilden, DE). Cells were permeabilized with 0.2% Triton/DEPC‐PBS for 10 minutes and dehydrated with 70%, 90% and 100% ethanol and desiccant chamber. After being air‐dried, a pre‐hybridization with 50% formamide/50 mmol/L sodium phosphate buffer/10% dextran sulphate/2X saline‐sodium citrate (SSC) buffer for 1 hour at 66°C was performed. The probe was denatured in hybridization buffer for 75 seconds at 80°C, and 40 nmol/L was added to 200 μL of pre‐hybridization buffer for each slide. Hybridization was performed in a humidified chamber for 20 hours at 66°C. Slides were washed once in 2X SSC/0.1% Tween 20 and then three times in 0.1X SSC before being ethanol dehydrated and mounted with ProLong Gold anti‐fade reagent with DAPI. Images were acquired by confocal microscope and analyses of foci performed with ImageJ software.

### 2.9 Southern Blot

For Southern Blot (SB) analysis, a ^32^P‐radioactive *C9ORF72* probe 466bp‐long up‐stream the repeat expansion was obtained with the following primers: FOR: 5′CTTTCTCCAGATCCAGCAGCCTCC3′ and REV: 5′CTGAGTTCCAGAGCTTGCTACAG3′. A total amount of 9 μg of genomic DNA was digested overnight at 37°C with 50 U of XbaI restriction enzyme and 1% BSA. The day after, digested DNA and molecular weights (MW 1 kb and XV) were loaded on a 0.7% agarose gel in TBE and electrophoresed for 2 hours at 70 V and overnight at 40 V. On the third day, gel was washed in HCl 0.25 N, gently shaken for 20 minutes to break the DNA into smaller fragments and enhance the transfer onto hybridization membrane. DNA was then transferred to a positively charged nylon membrane (Amersham Hybond‐N+, GE Healthcare) by capillary blotting overnight and cross‐linked by UV irradiation. After pre‐hybridization at 42°C for 6 hours, hybridization with the specific probe was carried out at 42°C overnight. The membrane was then washed in 2XSSC/0.1% sodium dodecyl sulphate (SDS) at room temperature for 20 minutes and in 0.5X SSC/0.1% SDS at 60°C for 15 minutes. After a brief wash in SSC 0.1X, the dried blot was placed in the film cassette with the auto‐radiographic film and exposed for at least two nights at −20°C before developing.

### 2.10 Sodium arsenite treatment and stress granules evaluation

MNs were seeded on coverslips at a concentration of 10 × 10^5^ cells/well and on day 10 exposed to 0.5 mmol/L sodium arsenite (Merck). After 1 hour of treatment, cells were fixed for immunocytochemistry. The presence of stress granules was revealed by immunofluorescence as previously described using a specific anti‐TIAR‐1 (1:300, Cell Signaling Technology) antibody.

### 2.11 Quantification and statistical analyses

Bar charts represent the mean ± standard error mean (SEM). Statistical differences were assessed by Fisher's exact test and *t* test. A *P* value of <.05 was considered significant.

## 3 RESULTS

### 3.1 No differences were observed between FB‐ and PB‐derived iPSCs regarding their reprogramming potential and stemness

**Figure 1:**
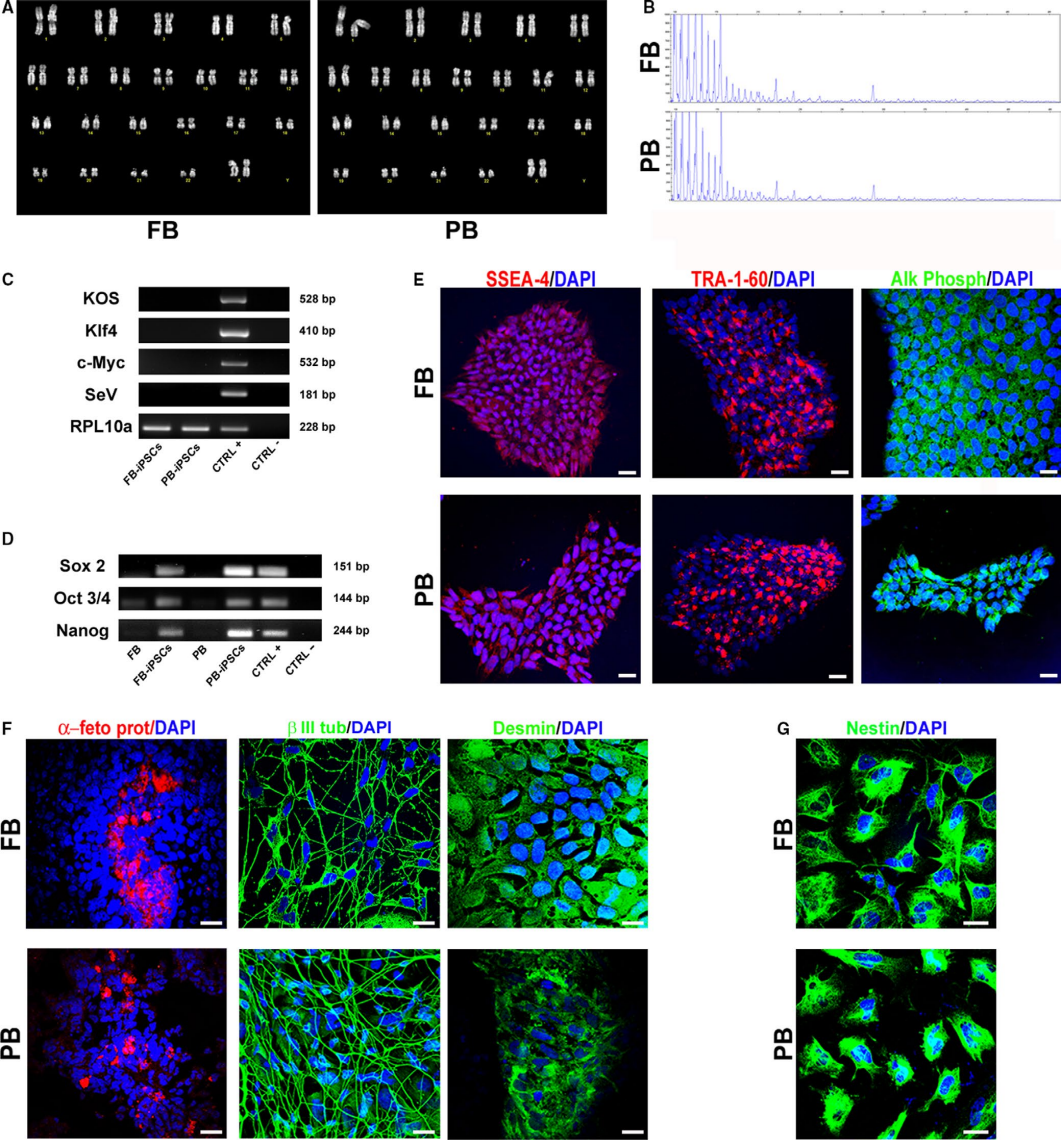
Characterization of induced pluripotent stem cells (iPSCs). A, Q‐banding karyotype of FB and PB‐derived iPSCs. B, Repeat primed PCR of iPSCs from FB and PB to confirm the G_4_C_2_‐repeat expansion. C, RT‐PCR analysis for the transgene KOS, Klf4, c‐Myc and SeV performed on FB‐iPSCs and PB‐iPSCs after the fifth passage. Reprogrammed FB were used as positive control. RPL10a was used as loading control. D, Expression of iPSC specific markers Sox 2, Oct 3/4 and Nanog assessed by RT‐PCR on starting tissues (FB and PB), iPSCs and positive control (other iPSC line). E, Representative images of iPSC colonies derived from FB and PB stained for the stemness markers SSEA‐4, TRA‐1‐60 and alkaline phosphatase (Alk Phosph). F, Differentiated EBs spontaneously generated cells of the three germ layers: endoderm (α‐feto protein positive), ectoderm (β III tubulin positive) and mesoderm (desmin positive). G, Representative images of iPSC lines differentiated towards neural fate. iPSCs differentiated for 7 days in induction medium and expanded for five passages in expansion medium became nestin positive. Nuclei were counterstained with DAPI. Scale bars, 100 μm. Images are representative of 1‐2 independent experiments

We found no differences in the reprogramming efficiency comparing the two different cell sources. Both cell types gave rise to more than 12 emerging colonies after reprogramming and 6 clones for each source were picked and showed the same expansion rate. iPSCs clones from both tissues formed compact colonies, presenting cells with high nucleus/cytoplasm ratio and distinct colony border. Colonies were able to be maintained over 50 passages and transgene vectors were lost within the first five passages, as shown in Figure 1C. iPSCs were subjected to a standard cytogenetic procedure to ascertain their normal karyotype (Figure 1A), and the maintenance of the *C9ORF72* expansion was established by repeat primed PCR (Figure 1B).

To ensure their stemness, expression of specific intracellular markers was evaluated by RT‐PCR (Sox 2, Oct 3/4 and Nanog) and by immunofluorescence (SSEA‐4, TRA‐1‐60 and alkaline phosphatase). Expression of early transcription factors, absent or weakly detectable in starting tissues, confirmed staminality of our iPSCs (Figure 1D). Additionally, both samples showed positivity for the expression of stemness proteins (Figure 1E). We conducted pluripotency assays of the clones, assessing their ability to spontaneously differentiate into cells of the three germ layers. iPSCs formed spherical embryoid bodies (EBs) when cultured in suspension for 7 days in non‐adherent dishes. Once allowed to adhere on Matrigel‐coated dishes, EBs from both clones spontaneously differentiated into endodermal (α‐fetoprotein positive), ectodermal (β III tubulin positive) and mesodermal (positive for Desmin) cells (Figure 1F). Furthermore, if cultured in a specific neural induction medium, iPSCs changed their typical shape, becoming 100% positive for the neural stem/progenitor cell marker nestin (Figure 1G) either way.

Given these data, during this preliminary characterization phase, no differences were observed between clones in terms of stemness properties, pluripotency and ability to differentiate into neural cells.

### 3.2 FB‐ and PB‐derived iPSCs displayed the same ability to differentiate into motor neurons

**Figure 2:**
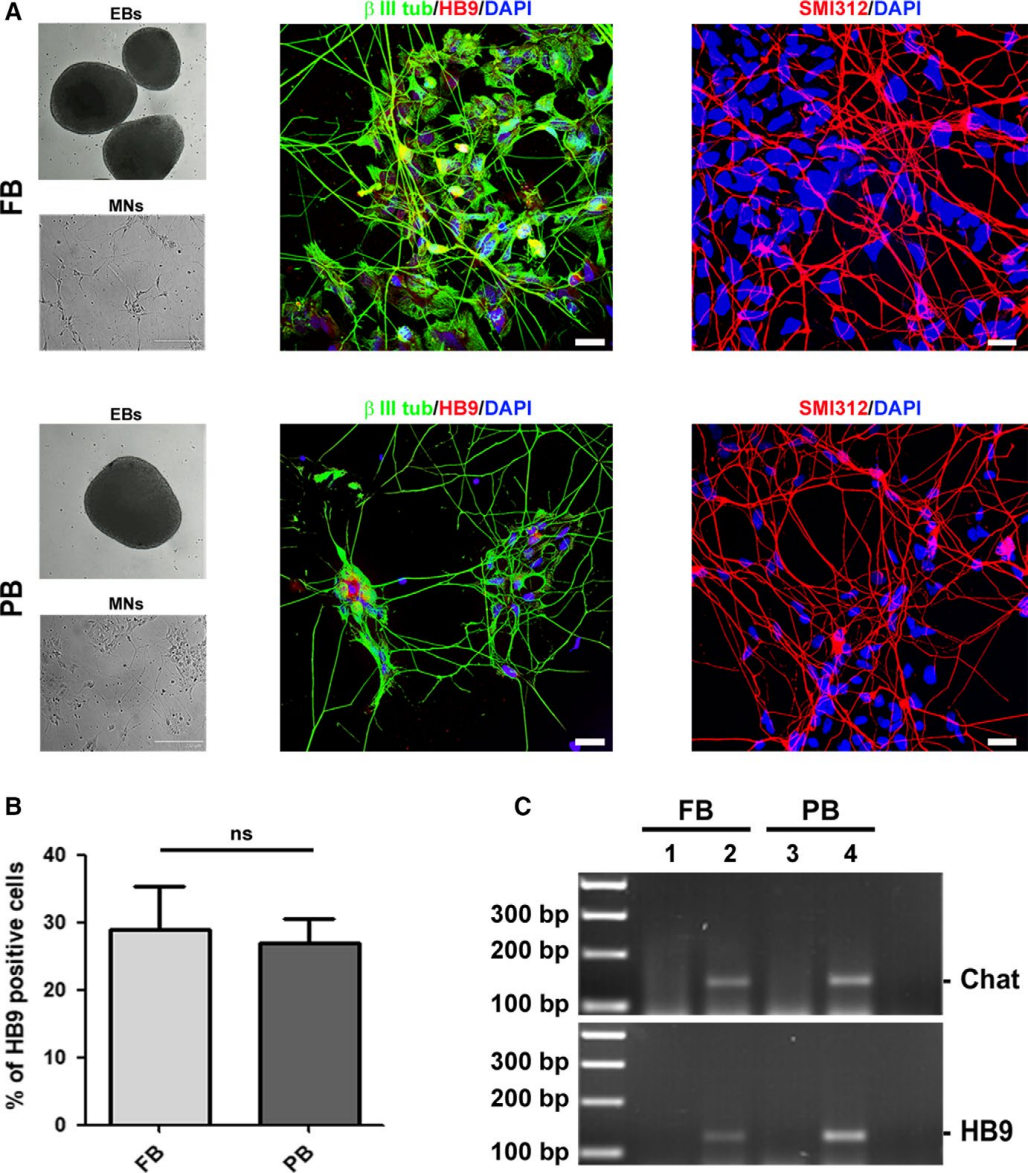
Comparison of *C9ORF72*‐mutated iPSC‐derived motor neurons. A, iPSCs from FB and PB were induced to generate embryoid bodies (EBs). After this first step, EBs were dissociated and differentiated into motor neurons (MNs). MNs were stained with the specific nuclear HB9 antigen (red) and co‐stained with the microtubule‐specific marker β III tubulin (green). The positivity for the pan‐axonal neurofilament marker SMI312 was also assessed (red). Nuclei were counterstained with DAPI. Scale bar, 20 µm. B, Histogram displaying the percentage of HB9 positive cells in iPSC‐derived MNs from FB and PB. C, Expression of MNs‐specific markers Chat and HB9 evaluated by RT‐PCR on both iPSCs (lanes 1 and 3) and iPSC‐derived MNs (lanes 2 and 4). Images are representative of 1‐2 independent experiments

Upon cells characterization, in a second set of experiments we proceeded in evaluating the capability of one iPSC clone derived from FB and PB to differentiate into motor neurons (MNs). Cells were induced to differentiate into MNs within 10 days and then characterized by RT‐PCR and immunocytochemistry. Regardless of the cell source, iPSCs displayed the same ability to form EBs and to give rise to MNs (Figure 2A). Cells were stained to verify the positivity for different specific markers such as β III tubulin (neuronal microtubules), HB9 (MNs) and SMI312 (neurofilaments, pan axonal) (Figure 2A). All cells showed positive staining for β III tubulin and SMI312, while HB9 was present in a lower but comparable percentage of cells in both samples. In particular, an average number of 328 cells from two independent experiments was analysed, determining that the percentage of HB9 positive MNs was 29% (±14) and 26.9% (±8.3) for FB and PB, respectively (Figure 2B) (*P* value .79). Moreover, HB9 and choline acetyltransferase (Chat) mRNAs were expressed in both MN samples after differentiation but not in iPSCs, as revealed by RT‐PCR (Figure 2C), confirming a successful neuronal commitment.

### 3.3 C9ORF72 mutation‐specific hallmarks were present in the same way in both iPSCs and iPSC‐derived MNs

**Figure 3:**
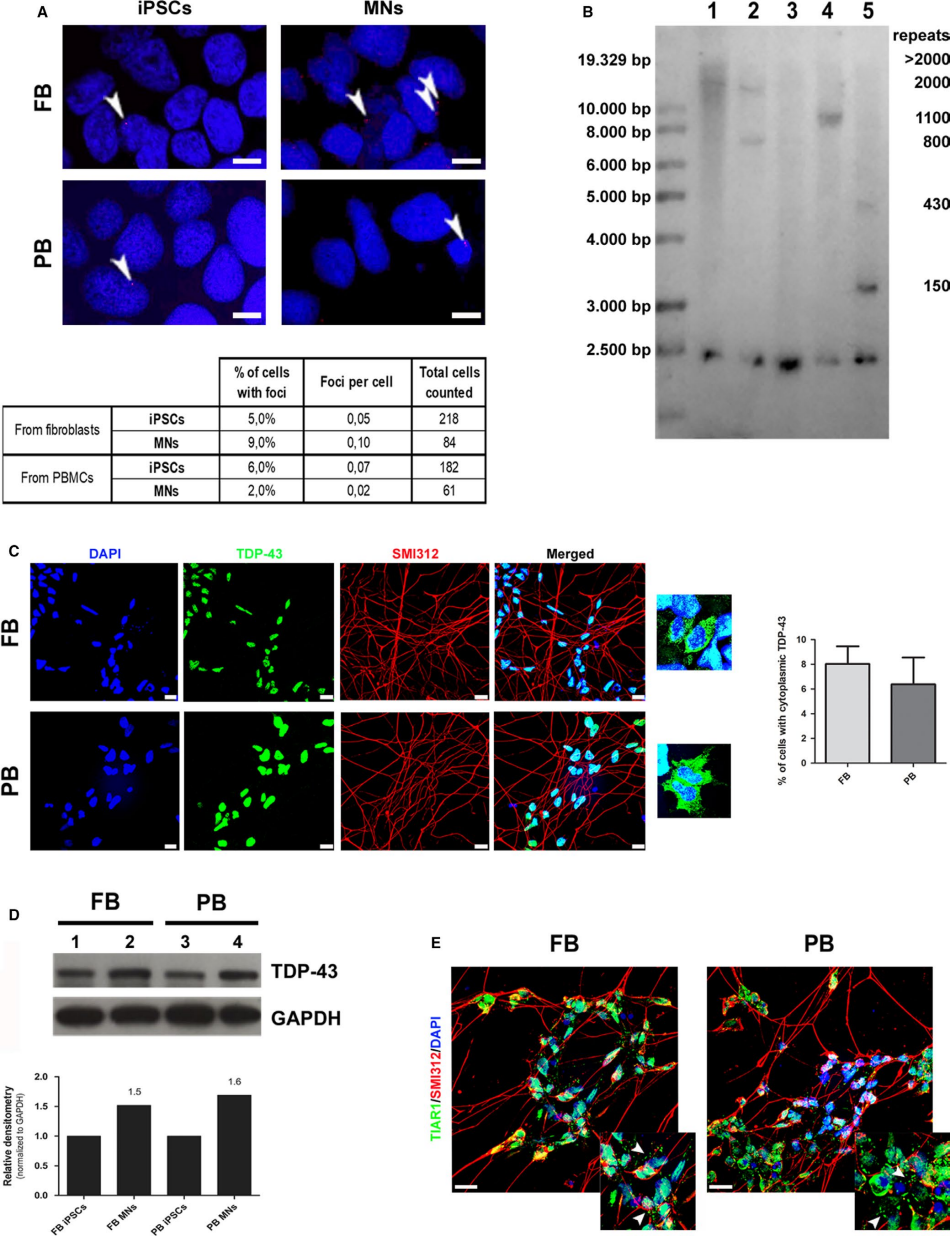
Comparison of specific pathological hallmarks in FB‐ and PB‐derived cells. A, The percentage of cells with foci and the number of foci per cell were evaluated in both iPSCs and iPSC‐derived MNs by FISH technique. Foci are indicated by head arrows. Scale bar, 5 µm. B, Southern blot analysis on DNA obtained from PB and FB (lanes 1 and 4) and PB‐ and FB‐derived iPSCs (lanes 2 and 5). Sample from a healthy donor (negative control) is shown in lane 3. C, Motor neurons derived from FB‐ and PB‐iPSCs were analysed to observe localization and aggregation of TDP‐43 protein (green) by immunofluorescence. Cells were co‐stained with the pan‐axonal neurofilament marker SMI312 (red). Scale bar, 20 µm. Magnification of cells displaying a cytoplasmic mislocalization. D, Western blot analysis of the total amount of TDP‐43 protein in iPSCs (lanes 1 and 3) and iPSC‐derived MNs (lanes 2 and 4). Densitometry analysis in lower panel: for both FB and PB‐derived MNs, the expression was calculated using as reference the TDP‐43 protein level of their corresponding iPSCs. E, Representative images of differentiated MNs following acute treatment with sodium arsenite (0.5 mmol/L, 1 h). After treatment, MNs were immunostained for the stress granule marker, TIAR1 (green) and the pan‐axonal neurofilament marker SMI312 (red). Scale bar, 20 µm. Stress granules are indicated by head arrows in the magnification panels. MNs images are representative of two independent experiments

We subsequently investigated some specific hallmarks of the *C9ORF72* mutation. In particular, it is known that the presence of the G_4_C_2_‐repeat expansion leads to the formation of nuclear and cytoplasmic RNA foci constituted by sense and antisense mutant intronic RNA. We confirmed the presence of cells forming sense foci in our iPSCs and iPSC‐derived MNs, evaluating by fluorescence in situ hybridization (FISH) the number of cells forming foci and the number of foci per cell. Again in both samples analysed, no significant differences were observed neither in the percentage of cells showing foci nor in the number of foci per cell (Figure 3A). Fisher's exact test statistic value is 0.1827 and 0.0894, respectively.

Variation of G_4_C_2_‐repeat length can be detected among different tissues of the same individual. We found that the repeat size, evaluated by Southern blot (SB) analysis, was originally different in PB and FB (Figure 3B, lane 1 and 4, respectively) of our patient, suggesting that intrinsic properties of specific tissue might affect the stability of the expanded repeats. Furthermore, once reprogrammed, we observed a decrease in the number of G_4_C_2_‐repeats in both PB‐ and FB‐derived iPSCs (Figure 3B, lane 2 and 5, respectively) compared with the starting cells, remaining however within a range of pathological size (higher than 30 repeats). Moreover, SB analysis revealed the presence of two different bands in both FB‐ and PB‐derived iPSCs, with different sizes (430 and 150 repeats for FB; 2000 and 800 repeats for PB).

### 3.4 A prevalent nuclear localization of TDP‐43 protein is shown in both iPSC‐derived MNs

The presence of cytoplasmic aggregates of the TDP‐43 protein is another common neuropathological hallmark, even if FB and iPSCs derived from ALS and FTD patients do not display the characteristic aggregates observed in affected brains. To deepen our characterization, we investigated the presence and the localization of TDP‐43 protein. Immunocytochemistry of both iPSC‐derived MNs revealed that TDP‐43 was localized almost totally within the nucleus with few cells displaying cytoplasmic mislocalization of the protein. In particular, an average number of 483 cells from two independent experiments was analysed, determining that the percentage of iPSC‐derived MNs displaying a cytoplasmic mislocalization of TDP‐43 was 8% (±2.9) and 6.22% (±3.4) (*P* value .56) for FB and PB, respectively (Figure 3C). Similarly, neither mislocalization nor aggregates were observed using an anti‐phospho TDP‐43 antibody (data not shown).

On the other hand, the total amount of TDP‐43, evaluated by Western blot (WB), was comparable between FB‐ and PB‐derived iPSCs and between iPSC‐derived MNs of both tissues. Higher levels of the protein were observed in MNs vs iPSCs in both models (1.5 and 1.6‐fold increase for FB and PB, respectively) (Figure 3D).

### 3.5 iPSC‐derived MNs produce stress granules following acute oxidative stress treatment

ALS‐related proteins are also involved in stress granules dynamics, a highly adaptive mechanism built up in response to stressful environmental insults. To mimic acute stress conditions in vitro*,* we treated iPSC‐derived MNs at day 10 of differentiation with 0.5 mmol/L sodium arsenite for 1 hour and analysed cell responses. Following acute exposure to sodium arsenite, both iPSC‐derived MNs were able to produce TIAR positive stress granules in the 100% of the cells as adaptive mechanism (Figure 3E, with magnification panels). No differences were found in terms of number of stress granules formed by both samples (average number of 2.5 granules/cell for FB vs 2.2 granules/cell for PB, 80 cells/sample analysed).

## 4 DISCUSSION

This is a proof‐of‐principle study aimed to compare FB‐ and PB‐derived iPSCs from the same individual.

In particular, our aim was to investigate the key aspects of iPSCs and iPSC‐derived MNs obtained from both FB and PB of a *C9ORF72*‐mutated patient to establish their comparability. Differentiation of human neural progenitor cells from iPSCs has been already demonstrated to occur similarly regardless their somatic tissue origin.^14^ However, a comparison of iPSC‐derived MNs from two tissues of the same *C9ORF72*‐mutated ALS patient has never been performed. The *C9ORF72* mutation was chosen in this study as the most common genetic cause of ALS and FTD,^15^, ^16^ two fatal neurodegenerative diseases. In addition, patient cells carrying this mutation exhibit well known specific pathogenic molecular hallmarks that can be explored, such as RNA foci and repeat expansion length. In our work, initial characterization studies of iPSCs showed no differences between the clones investigated in terms of stemness properties, markers expression, pluripotency and ability to differentiate into neural cells demonstrating that iPSCs obtained from FB and PB show equivalent characteristics. This was also true for the iPSCs differentiation into MNs. Clones displayed the same ability to differentiate into β III tubulin/SMI312 positive MNs, presenting a similar percentage of HB9 expressing cells.

The presence of the G_4_C_2_‐repeat expansion in the noncoding region of the *C9ORF72* gene leads to a *C9ORF72‐*reduced expression, suggesting haploinsufficiency as one of the potential disease‐causing mechanisms.^17^ Additional specific pathological hallmark of *C9ORF72* patients is represented by the presence of nuclear and cytoplasmic RNA foci formed by sense and antisense G_4_C_2_‐repeat transcripts^12^, ^18^ as well as accumulation of five different dipeptide repeat proteins (DPRs) generated by mean of a repeat‐associated non‐AUG (RAN) translation, mostly in the neuron cytoplasm.^19^ Previous reports have already demonstrated the presence of both RNA foci and DPRs in *C9ORF72*‐mutated iPSC‐derived MNs.^20^, ^21^ In this study, we quantified the number of cells showing foci and also the number of foci per cell in our iPSCs and iPSC‐derived MNs without finding statistically significant differences among all samples.

The G_4_C_2_‐repeat expansion was confirmed by both primed PCR and SB analyses in our FB‐ and PB‐derived iPSCs. In this study, not surprisingly, the two tissues displayed different sizes of the repeat expansion. Indeed, intra‐individual dissimilarity of number of repeats between tissues has already been described.^22^ In addition, after reprogramming, we observed a contraction in the size of the expansion in both samples. It is conceivable that this contraction, probably caused by reprogramming, is due to a selection of clones with a less extended repeat length and therefore higher stability. Furthermore, SB analysis revealed the presence of two expansions of different sizes in both iPSC samples. Considering the clonal origin of iPSCs, this result appears unexpected and could reflect a mosaicism that probably occurred during expansion of iPSCs. In fact, instability of expanded repeats depending on the passage number of iPSCs has already been reported for iPSCs derived from a patient affected by myotonic dystrophy type I, another disease caused by expanded repeat.^23^ The presence of clones with different sizes of expansion should be taken in consideration for further applicative studies.

Regarding another hallmark of ALS pathology, no pathological protein aggregates but a slight cytoplasmic mislocalization of the TDP‐43 protein were observed, in contrast to what is found in *post mortem* ALS patient MNs. Nevertheless, similar discrepancies between in vivo and in vitro cells were already been reported.^13^ Results from previous studies investigating the presence of TDP‐43 protein pathological phenotype in iPSC‐derived MNs vary considerably from one to the other. Burkhardt et al^24^ identified nuclear aggregates in 3 out 24 reprogrammed skin fibroblasts from sporadic ALS patients. Similarly, two authors reported cytoplasmic pre‐inclusion‐like aggregates on MNs derived from TDP‐43‐mutated patients^25^, ^26^ while no aggregation was observed in two other studies.^13^, ^27^ No subcellular localization was observed for TDP‐43 protein in iPSCs^20^ and iPSC‐derived MNs^21^ from *C9ORF72*‐mutated patients while nuclear depletion and cytoplasmic accumulation of the protein were reported by Zhang et al.^28^ The total amount of the TDP‐43 protein evaluated by WB was comparable between FB‐ and PB‐derived iPSCs and between iPSC‐derived MNs of both tissues, with a slight increase in MNs. This can be probably due to a higher metabolism and protein translation of the differentiated cells if compared to iPSCs.

No differences were found in the response to acute stress provoked by sodium arsenite treatment. Both iPSC‐derived MNs were able to produce TIAR positive stress granules as adaptive mechanism to stressful environmental insults.

## 5 CONCLUSION

iPSCs and iPSC‐derived MNs represent powerful patient‐specific models to investigate pathological mechanisms in diseases such as ALS and FTD. In this study, for the first time, we demonstrated that reprogrammed cells from PB are fully comparable with reprogrammed FB, underling that the cell source does not affect iPSC peculiarities, differentiation potential and specific pathological features. For some diseases, such as rare or childhood pathologies, different types of tissue may be available from different patients and we have successfully demonstrated that the resulting iPSCs can be compared anyway. Our findings represent new evidence, leading to develop promising tools for future translational and therapeutic applications.

## CONFLICT OF INTEREST

The authors confirm that there are no conflicts of interest.

## AUTHOR CONTRIBUTIONS

DB performed the research, analysed the data and wrote the paper. FS, CC, CV, VG performed part of the experiments. SP performed the primed PCR and IC the karyotypes. AR provided the samples. VS provided the samples, the clinical data and supervised the all study. PB designed the research study, performed part of the experiments, analysed the data, wrote the paper and supervised the all study. All authors read and approved the final manuscript.

## Data Availability

The data that support the findings of this study are available from the corresponding author upon reasonable request.
