# Supplementary material for: Reprogramming fibroblasts and peripheral blood cells from a C9ORF72 patient: A proof‐of‐principle study
Source: J Cell Mol Med. 2020 Mar 3;24(7):4051–60. doi: 10.1111/jcmm.15048 (PMC7171411; doi:10.1111/jcmm.15048)
Supplement: Supplementary file 1 [file JCMM-24-4051-s001.docx]

**Supplementary KEY RESOURCES TABLE**

| **REAGENT or RESOURCE** | **SOURCE** | **IDENTIFIER** |
| --- | --- | --- |
| **Antibodies** | | |
| SSEA-4 | Invitrogen | 14-8843-80 |
| Tra-1-60 | Invitrogen | 14-8863-80 |
| Alkaline Phosphatase | Abcam | ab108337 |
| Desmin | Chemicon | ab907 |
| B III Tubulin | Abcam | ab52623 |
| Alfa-fetoprotein | Invitrogen | 14-6583-80 |
| SMI312 | Covance | SMI-312 |
| Nestin | Chemicon | MAB5326 |
| TDP-43-2AP | Proteintech | 10782-2-AP |
| Phospho TDP-43 (pS409/410) | Cosmobio | TIP-PTD-M01 |
| HB9 | DSHB | 81.5C10 |
| TIAR-1 | Cell Signaling Technology | 8509S |
| GAPDH | Santa Cruz | SC-32233 |
| Anti-mouse HRP-conjugated | Santa Cruz | SC-2005 |
| Anti-rabbit HRP-conjugated | Santa Cruz | SC-2004 |
| Alexa Fluor 555 anti mouse | Invitrogen | A21422 |
| Alexa Fluor 488 anti mouse | Invitrogen | A11017 |
| Alexa Fluor 488 anti rabbit | Invitrogen | A11070 |
| **Biological Samples** |  |  |
| Fibroblasts from a C9orf72 mutated ALS patient | This paper |  |
| Peripheral blood from a C9orf72 mutated ALS patient | This paper |  |
| iPSCs from a C9orf72 mutated ALS | This paper |  |
| iPSC-derived motor neurons from a C9orf72 mutated ALS | This paper |  |
| **Chemicals, Peptides, and Recombinant Proteins** | | |
| Histopaque®-1077 | Sigma-Aldrich | H8889 |
| Retinoic Acid | Sigma-Aldrich | R2625 |
| Poly-L-lysine | Sigma-Aldrich | P0899 |
| Laminin | Sigma-Aldrich | L2020 |
| bFGF | Peprotech | 100-18C |
| Purmorphamine | Sigma-Aldrich | SML0868 |
| LDN-212854 | Sigma-Aldrich | SML0965 |
| Glutamate | Sigma-Aldrich | G8415 |
| Y-27632 2HCL | SelleckChem | S1049 |
| SB 431542 | Tocris | 301836-41-9 |
| SAG | Tocris | 912545-86-9 |
| N2 | Gibco | 17502-048 |
| B27 | Gibco | 17504-044 |
| IL-3 | Gibco | PHC0034 |
| IL-6 | Gibco | PHC0064 |
| GDNF | Peprotech | 450-10 |
| CNTF | Peprotech | 450-13 |
| SCF | Gibco | PHC2115 |
| FLT-3 Ligand | Gibco | PHC9414 |
| BDNF | Peprotech | 450-02 |
| Matrigel | Corning (BD) | 354277 |
| AccuPrime GC-Rich DNA Polymerase | Invitrogen | 12337016 |
| 7-Deaza-2′-deoxy-guanosine-5′-triphosphate | Roche | 10988537001 |
| dNTP Set 100 mM Solutions | Thermo Fisher Scientific | R0181 |
| Hi-Di Formamide | Applied Biosystems | 4311320 |
| GeneScan 500 ROX dye Size Standard | Applied Biosystems | 401734 |
| Trypsin-EDTA solution | Sigma-Aldrich | T4049 |
| KnockOut Serum Replacement | Thermo Fisher Scientific | 10828028 |
| Normal Goat Serum | Gibco | 16210064 |
| Sodium Arsenite solution | Merck | 1.06277.1000 |
| Quinacrine Mustard | Sigma-Aldrich | Q2876 |
| Accutase | Euroclone | ECB3056D |
| **Critical Commercial Assays** | | |
| CytoTune™-iPS 2.0 Sendai Reprogramming Kit | Thermo Fisher Scientific | A16517 |
| Wizard® Genomic DNA Purification Kit | Promega | A1125 |
| Pierce BCA protein assay | Thermo Fisher Scientific | 23225 |
| **Oligonucleotides** | | |
| Sox 2 FW - TTGCGTGAGTGTGGATGGGATTGGTG | Eurofins |  |
| Sox 2 RV - GGGAAATGGGAGGGGTGCAAAAGAGG | Eurofins |  |
| Oct 3/4 FW - GACAGGGGGAGGGGAGGAGCTAGG | Eurofins |  |
| Oct 3/4 RV - CTTCCCTCCAACCAGTTGCCCCA AAC | Eurofins |  |
| Nanog FW - CAGCCCTGATTCTTCCACCAGTCC | Eurofins |  |
| Nanog RV - GTTCTGGAACCAGGTCTTCACCT G | Eurofins |  |
| Chat FW - TGAGTACTGGCTGAATGACATG | Eurofins |  |
| Chat RV - AGTACACCAGAGATGAGGCT | Eurofins |  |
| HB9 FW - GCACCAGTTCAAGCTCAAC | Eurofins |  |
| HB9 RV - GCTGCGTTTCCATTTCATCC | Eurofins |  |
| FAM FW - TGTAAAACGACGGCCAGTCAAGGAGGGAAACAACCGCAGCC | Eurofins |  |
| Reverse primer - CAGGAAACAGCTATGACCGGGCCCGCCCCGACCACGCCCCGGCCCCGGCCCCGG | Eurofins |  |
| Reverse Anchor M13 primer - CAGGAAACAGCTATGACC | Eurofins |  |
| KOS FW - ATGCACCGCTACGACGTGAGCGC | Thermo Fisher Scientific |  |
| KOS RV - ACCTTGACAATCCTGATGTGG | Thermo Fisher Scientific |  |
| Klf4 FW - TTCCTGCATGCCAGAGGAGCCC | Thermo Fisher Scientific |  |
| Klf4 RV - AATGTATCGAAGGTGCTCAA | Thermo Fisher Scientific |  |
| c-Myc FW - TAACTGACTAGCAGGCTTGTCG | Thermo Fisher Scientific |  |
| c-Myc RV - TCCACATACAGTCCTGGATGATGATG | Thermo Fisher Scientific |  |
| SeV FW - GGATCACTAGGTGATATCGAGC | Thermo Fisher Scientific |  |
| SeV RV - ACCAGACAAGAGTTTAAGAGATATGTATC | Thermo Fisher Scientific |  |
| RPL10a FW - CAAGAAGCTGGCCAAGAAGTATG | Eurofins |  |
| RPL10a RV - TCTGTCATCTTCACGTGAC | Eurofins |  |
| **Software and Algorithms** | | |
| GraphPad Prism 5.0 | GraphPad Software Inc. | http://www.graphpad.com/scientific-software/prism/ |
| Gene Mapper v.4 | Applied Biosystem |  |
| ImageJ | NIH | https://imagej.nih.gov/ij/ |
| **Other** | | |
| Amersham Hybond-N+ | GE Healthecare | RPN303B |
| XbaI | New England Biolabs | R0145S |
| NuPAGE Novex 4-12% Bis-Tris Protein Gels | Thermo Fisher Scientific | NP0335BOX |
| iBLOT Gel Transfer Stacks Nitrocellulose | Thermo Fisher Scientific | IB301002 |
| Clarity Western ECL Substrate | BioRad | 170-5060 |
